# Sudden Cardiac Death Risk Perception and Its Relation to Personal Lifestyle Among Female University Students During the COVID-19 Pandemic

**DOI:** 10.7759/cureus.26255

**Published:** 2022-06-23

**Authors:** Samah F Ibrahim, Mai S Alharbi, Malath A Alrowili, Samiyah A Alaswad, Taghreed A Haidarah, Ghada A Alharbi, Amel Fayed

**Affiliations:** 1 Clinical Sciences, Princess Nourah bint Abdulrahman University, Riyadh, SAU; 2 Forensic Medicine, Cairo University, Giza, EGY; 3 Clinical Sciences, Princess Nourah Bint Abdulrahman University, Riyadh, SAU; 4 College of Medicine, Princess Nourah Bint Abdulrahman University, Riyadh, SAU

**Keywords:** survey, lifestyle change, saudi university students, risk perception, sudden cardiac death

## Abstract

Introduction

Risk perception is the key component of many health behavior changes. This study identified the deliberative sudden cardiac death (SCD) risk perception among young females during the coronavirus disease 2019 (COVID-19) pandemic and its implication on their willingness to lifestyle change in the Riyadh region, Saudi Arabia. This cross‑sectional study using self‑administered online questionnaires was conducted to reach a total of 797 female university students in Riyadh, Saudi Arabia.

Results

Eighty-six percent of participants showed moderate SCD risk perception, with a mean score of 20.4±4.4. Ninety-six percent of participants had ≥1 established SCD risk factor. A family history of cardiovascular disease and SCD was the most commonly reported risk factor (75.5%), followed by physical inactivity (75.4%). Nearly 60% of participants showed a high willingness to change personal lifestyle behaviors, however, the presence of risk factors did not significantly enhance their willingness tochange in order to control these risk factors.

Conclusions

This study identifies the deliberative SCD risk perception among young Saudi women and raises the need for preventive health care programs that enhance healthy behaviors among students at high risk, to minimize cardiovascular diseases and fatalities.

## Introduction

Sudden cardiac death (SCD) is an unexpected death due to the sudden non-traumatic cardiac arrest of an apparently healthy individual [1]. The global incidence of SCD ranges between 50 and 100 cases/100,000 individuals in the general population [2-4]. The highest proportion of cardiac-related deaths in Gulf countries is found in Kuwait, then the United Arab Emirates, followed by Saudi Arabia (41%, 40%, and 37%, respectively) [5]. In the Eastern region of Saudi Arabia, 59% of SCDs have been diagnosed with cardiovascular pathology [6].

SCD risk factors include smoking, family history of cardiac (diabetes, dyslipidemia) and history of cardiovascular diseases, and/or non-cardiac illnesses (hypertension and obesity) [7]. Moreover, several drugs used in the treatment of neuropsychiatric disorders are associated with an increased risk [8]. The newly emerged coronavirus disease 2019 (COVID-19) virus had an adverse effect on both the mental as well as physical health of individuals, with an increased number of reported SCD cases [9-10].

The incidence increases with age, however, the percent of sudden deaths without preexisting cardiac illness is more identified among females of younger age [11]. Saudi females aged 15-34 years represent 18% of the Saudi population. Unmarried females represent 6.2% while 5.2% of Saudi females are suffering from chronic diseases [12].

Studies have been conducted to assess women’s awareness of cardiovascular diseases and their related deaths [5,13-15], however, there is a lack in the link between SCD risk perception and adherence to preventive behaviors. Like the Alruways study [5], AL-Tamimi and Bawazir also found that the perception of CVD susceptibility among Saudi females was low and the Saudi population needs well-organized awareness campaigns about cardiovascular disease risk factors as a preventive intervention measure [16].

Therefore, this study was designed to identify the deliberative SCD risk perception among young females during the COVID-19 pandemic and its implication on their lifestyle change intentions in Riyadh region, Saudi Arabia.

## Materials and methods

Study design

This cross-sectional survey was conducted in the period between September 2020 and May 2021 after obtaining ethical approval from the Institutional Review Board of Princess Nourah Bint Abdulrahman University in December 2020, with reference number 20-0504.

Previous literature estimated that the yearly incidence of SCD in young adults ranged between 0.8 and 2.8 per 100,000 persons. The approximate number of public university students in Riyadh city is 246512; 51.8% of them are female [17]. The minimal convenience sample size was estimated to be 724, with an expected difference as low as 10% when the level of confidence is 95% (alpha=0.05), and the power of study of 80% (beta=20%); as a result, the researchers increased the sample size to 797 to compensate for uncompleted responses of 10%.

Data collection

A self-administered validated electronic questionnaire was prepared in the Arabic language on REDcap and posted online. The link was then distributed to the leaders of the students’ clubs of the principal Riyadh public universities through their social media accounts, and they were then asked to disseminate it to their colleagues during their studies. The link included researchers’ information, research objectives, and informed consent.

Data collection tool

A validated Arabic form of the cardiovascular disease (CVD) risk questionnaire [15,18] consisted of four sections as follows:

 -First: Socio-demographic data, including age, college specialty, and income.

- Second: Personal risk factors like smoking, hypertension, diabetes, dyslipidemia, obesity, no regular exercise, drug addiction, history of cardiac disease, family history of cardiac diseases, and sudden cardiac death.

-Third: Perceived risk of a sudden fatal cardiac event (consists of 8 questions that had a 5-point Likert scale ranging from strongly disagree (0) to strongly agree (5); with a maximum score of 40. A score of 13 or less is considered low risk, 14-26 moderate risk, and 27 and above is a high-risk score) [18].

-Fourth: Intentions to change (perceived readiness for change in exercise, diet, and smoking habits; it comprised nine questions, three for each item. It had a 5-point Likert scale ranging from strongly disagree (0) to strongly agree (5). A score of 5 or less is considered a low perceived readiness, 6-10 a moderate perceived readiness, and 11 and above is a high perceived readiness [18].

The response rate, the percentage of completed responses in relation to the total received questionnaires, was 98%.

Statistical analysis

The Statistical Package for the Social Sciences (SPSS) software, version 22.0 (IBM Corp., Armonk, NY), was used for data analysis. Cronbach’s alpha, which was used to test the questionnaire’s reliability, was 0.85. Numerical variables were presented as mean (SD), and categorical variables were presented as numbers (percent). A comparison was performed using the chi-square and Kruskal-Wallis tests for qualitative variables and the t-test for quantitative data. A P-value of less than 0.05 was considered statistically significant. Pearson correlation was used to detect the correlation between scale, ordinal variables, and different means of question groups, whereas a chi-square test was used for categorical variables. The response rate, the percent of the completed responses in relation to the total received questionnaires, was 98%.

## Results

Sociodemographic characteristics

A total of 797 female university students participated in the study, with a mean age of 21.4±5 years; 68.3% of which were 21 years of age or below and 57.1% were in a non-health-related college. Almost all participants had an income ranging from moderate (43%) to high (52.8%) (Table 1).

**Table 1:** Sociodemographic characters and risk factors among participants *multiple answers; BMI: body mass index

Variable	Number (%)
Age-class	
≤21	552(69.3)
≥22	245(30.7)
Academic specialization	
Health	342(42.9)
Non-health	455(57.1)
Income	
Enough and save	421(52.8)
Enough	343(43)
Not enough	32(4)
Risk factors	
Positive family history	602 (75.5) *
No regular exercise	601(75.4)
Obesity (BMI≥ 30)	71(8.9)
Dyslipidemia	42(5.3)
Substance use	33(4.1)
Current smoking	19(2.4)
Heart disease	29 (3.6)
Hypertension	17(2.1)
Diabetes	13(1.7)

Personal risk factors

**Figure 1:**
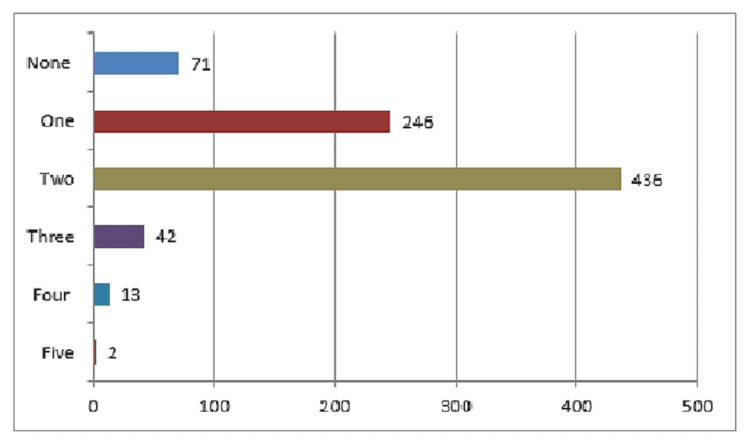
Number of cardiovascular disease and sudden cardiac death risk factors among participants

The mean number of positive risk factors per student was 1.65±.75 (Figure 1). A family history of CVD (75.5% SCD and 75.4% no regular exercise) was the highest reported risk factor, followed by obesity (8.9%), dyslipidemia (5.3%), and the presence of CVDs (3.6%). The least reported were diabetes (1.7%) and hypertension (2.1%). Substance use and smoking were reported by 4.1% and 2.4%, respectively. CVDs, including cardiac arrhythmia (premature ventricular contractions, sinus tachycardia) (65.5%), heart failure (20.7%), and myocardial infarction (13.8%), were reported in 29 students (Table 1).

**Figure 2:**
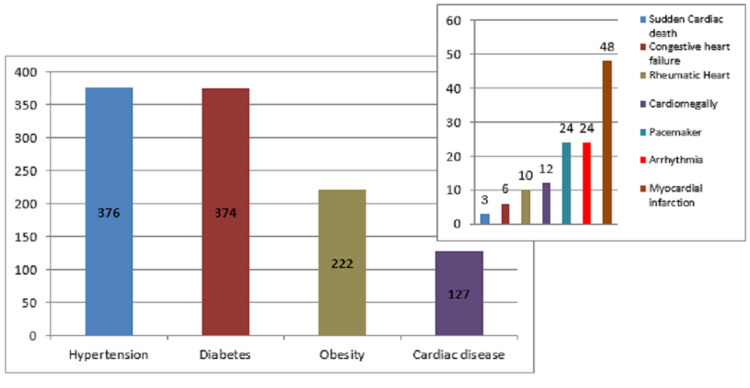
The frequency of family history of cardiovascular risk factors among participants

A total of 602 female university students had a family history of CVDs; 47.2% of which had hypertension and 46.9% had diabetes (Figure 2).

Regarding substance use, central nervous system (CNS) depressants, anabolic steroids, and CNS stimulants were reported by 2.5%, 1%, and 0.62%, respectively.

Regarding substance use, central nervous system (CNS) depressants, anabolic steroids, and CNS stimulants were experienced in 20, 8, and 5 participants respectively.

Perceived risk of SCD

The perceived risk of a sudden fatal cardiac event is assessed by eight questions that had a 5-point Likert scale ranging from strongly disagree (0) to strongly agree (5), with a maximum score of 40. A score of 13 or less is considered low risk, 14-26 a moderate risk, and 27 and above is a high-risk score.

The mean score of perceived SCD risk was 20.4±4.4, 86% of which were moderate while 7% of them were high as seen in Table 2.

**Table 2:** Responses to the perceived sudden cardiac death susceptibility, the perceived readiness to change sports, diet, and smoking behaviors

Number (%) Items	Strongly disagree	Disagree	Neutral	Agree	Strongly agree
Perceived sudden cardiac death susceptibility					
It is likely that I will suffer from a fatal heart attack in the future.	191(24)	273(34.3)	276(34.6)	48(6)	9(1.1)
My chances of suffering from a fatal heart attack in the next 10 years are great.	171(21.5)	312(39.1)	257(32.2)	48(6)	8(1)
It is likely I will have a fatal heart attack because of my past and/or present behaviors.	158(19.8)	260(32.6)	253(31.7)	102(12.8)	24(3)
I feel sure that I will have a fatal heart attack	295(37)	281(35.3)	192(24.1)	23(2.9)	6(.8)
I am concerned about the likelihood of having a fatal heart attack in the near future.	171(21.5)	199(25)	265(33.2)	126(15.8)	36(4.5)
I am not worried that I might have a fatal heart attack.	90(11.3)	147(8.4)	289(36.3)	161(20.2)	110(13.8
It is likely that if I suffer from a cardiac event it will be fatal.	124(15.6)	291(36.5)	244(30.6)	113(14.2)	25(3.1)
If I have a heart attack I will die within 10 years	48(6)	71(8.9)	327(41)	280(35.1)	71(8.9)
Total mean score	20.4±4.4				
Intention to change sports behaviors					
I am not thinking about exercising for 2½ hours a week.	52(6.5)	103(12.9)	158(19.8)	294(36.9)	190(23.8)
I am thinking about exercising at least 2½ hours a week.	32(4)	75(9.5)	160(20.1)	340(42.7)	189(23.7)
I am ready or have started to exercise 2½ hours a week.	73(9.2)	230(28.9)	221(27.7)	180(22.6)	93(11.7)
Total mean score					10.3±2.5
Intention to change diet behaviors					
I am not thinking about eating at least five portions of fruit and vegetables a day.	32(4)	135(16.9)	190(23.8)	269(33.8)	171(21.5)
I am thinking about eating at least five portions of fruit and vegetables a day.	33(4.1)	105(13.2)	184(23.1)	331(41.5)	144(18.1)
I am ready or started to eat at least five portions of fruit and vegetables a day.	72(9)	239(30)	250(31.4)	171(21.5)	65(8.2)
Total mean score					10±2.2
Intention to change smoking behaviors					
I am not thinking about stopping smoking.	4 (21)	5 (26.3)	4 (21)	4 (21)	2 (10.5)
I am thinking of stopping smoking within two months.	2(10.5)	5 (26.3)	3(15.8)	3(15.8)	6(31.6)
I have reduced or stopped smoking.	5(26.3)	2 (10.5)	6 (31.5)	5 (26.3)	1 (5.3)
Total mean score					8.8±1.9

Perceived readiness for change

**Figure 3:**
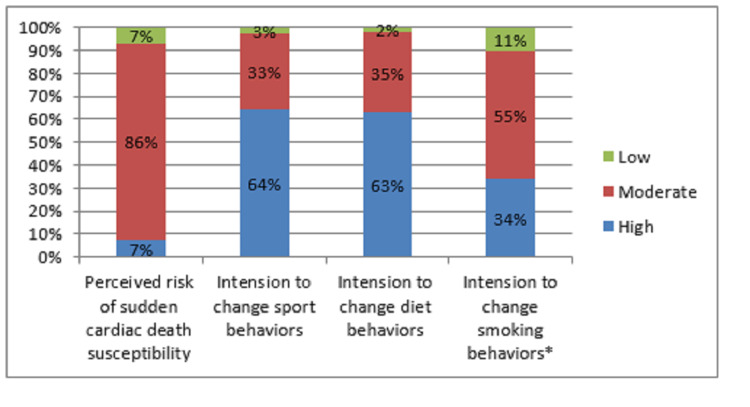
The scores of the perceived sudden cardiac death susceptibility and perceived readiness to change sports, diet, and smoking behaviors among participants *19 currently smokers

More than 60% of participants showed a high readiness for a change in their lifestyle behaviors, including physical activity and diet (Figure 3).

Regarding physical fitness, 66.4% were about to exercise at least 2½ hours a week while 43.3% had already started. Regarding diet behaviors, 59.6% of the participants thought about eating at least five portions of fruit and vegetables a day while 29.7% of them already started this behavior. As for diet, 59.6% were about to start a diet comprising at least five portions of fruit and vegetables a day while 29.7% had already started (Table 2).

Of the total number of smokers, 34% showed a high intention to change this habit (Figure 3), 47.4% considered changing this habit within two months while 31.6% had already reduced or stopped it (Table 2).

The correlation analysis declared the increasing number of cardiac risk factors was correlated significantly with increasing the perceived SCD (r=0.13) while it was noticed that the increasing number of cardiac risks negatively correlated with the students’ intention to improve their sports or diets habits (r=-0.11,- 0.12, respectively). However, perceived SCD was significantly lower among participants with a positive family history of cardiovascular disease (Table 3 and Table 4).

**Table 3:** The correlation between age, income, and risk factors with perceived sudden cardiac death susceptibility and intention for behavioral changes *significant P-value ≤0.05, the Spearman correlation coefficient was used

Items	Perceived sudden cardiac death susceptibility	Intention for behavioral changes		
		Sport	Diet	Smoking
Age	0.04	0.04	0.03	0.24)
Income ^a^	-0.015	-0.05	-0.02	0.13
Number of risk factors	0.13*	-0.112 *	-0.12 *	-0.24

**Table 4:** Associations between the participant's socio-demographic qualitative variables with perceived sudden cardiac death susceptibility (mean ± SE) and intention for behavioral changes (mean ± SE) *significant P-value≤0.05, # associations were tested among smokers

	Perceived sudden cardiac death susceptibility	Intention to change		
		Sport	Diet	Smoking^#^
Academic specialty				
Health	20.2±.2	10.3±.1	10.2±.1	7.7±.8
Non-health	20.6±.2	10.3±.12	10±.1	8.9±.3
Family history				
Yes	20±0.2*	10.3±0.14	10±0.13	7.8±1
No	21±0.3	10.6±0.2	10.4±0.16	10.5±.5
Having CVDs				
Yes	20.6±.8	10.3±.1	10.2±.4	8.8±.3
No	20.4±.2	10.2±.5	10±.08	8.7±.8

## Discussion

The perceived risk can enhance many health behavior changes [19]. Our results identified that perceiving moderate SCD risk among female university students enhanced their ability to adopt a healthy lifestyle in the current COVID-19 pandemic. These findings were in line with the Saudi study’s results that explored the perceived CVDs susceptibility among female Saudi teachers [15].

The number of the risk factors was positively correlated to the SCD perceived risk but positive family history showed a negative correlation with SCD perceived risk. Unfortunately, most of the variables, including education, or the presence of CVDs did not show any significant relation to SCD perceived risk. Together, these results suggested the need for university students, especially those having CVDs risk factors to gain more knowledge about SCD risk factors and skills to modify their life.

Another Saudi study found that physical inactivity, obesity, smoking, and family history of coronary artery disease among Saudis aged 18-25 years were associated with an increased CVD risk [5]. The reasons behind the increased burden of CVDs and SCD in the Gulf Cooperation Council countries, including Saudi Arabia, could be related to consanguineous marriage, economic development, and a sedentary lifestyle with unhealthy food consumption and physical inactivity [5,20].

Ninety-six percent of participants had ≥1 established SCD risk factor. The high perceived SCD risk scores were identified among 40.5%, 61.5%, and 100% of female students with 3, 4, and 5 risk factors, respectively. Aggregation of well-known SCD risk factors like age, increased body mass index, no regular exercise, nutritional facts, and chronic cardiovascular diseases, including irregular heart rate and familial incidence of SCD, could contribute to an increased SCD risk [21-22]. The lifetime risk of SCD will increase in individuals who develop several CVD risk factors at a younger age, including genetic predisposition.

Although modifiable risk factors, including smoking, hypertension, diabetes, hypocholesteremia, obesity, and behaviors related to an unhealthy lifestyle, including lower consumption of fruit and vegetables, and non-regular physical activity are major determinants of CVD morbidity and mortality [22], only 8.5% of those with these risk factors in our study reported a high perceived SCD risk. Moreover, the presence of these risk factors did not significantly encourage the young females to improve their lifestyle behaviors to control these modifiable risk factors.

Smoking was also one of the risk factors that significantly increased the SCD risk by three to fivefold [22]. This association should increase smokers’ concerns and motivate them to quit it [21].

According to Saudi Health Interview Survey, smoking prevalence was 12.1%, 1.5% of which were females [12]. In addition, smoking prevalence among university students ranged from 16.8-28.1%, 1.6% of which were females as compared to 2.4% in our study [5,23-24].

Generally, in Saudi Arabia, men tend to have higher smoking rates than females, yet gender variances in smoking behavior have diminished over time [25].

Although a significant effort has been made in high-income countries, including Saudi Arabia, to outline and modify CVDs’ risk factors, the incidence of CVDs continues to increase [26]. The continuously rising CVDs and SCD incidence could be attributed to the inadequate prevention of modifiable/preventable risk factors, including cigarette smoking, unhealthy diet, and physical inactivity. Therefore, we assessed the students’ intentions to modify these CVD-related lifestyle habits and its relation to their perceived SCD risk.

In this study, studying in health-related specialties, the smaller number of CVDs risk factors, and the absence of regular exercise significantly increased students’ intention to modify their physical activity and dietary behaviors.

A moderate to high intention to quit smoking was found among the majority of our smoker participants. We were in the same line with a Korean study that detected a high intention to quit smoking in women to improve their physical activity [27]. This finding raised the essential idea that smoking cessation can alter the SCD risk. The SCD risk among women is linearly reduced with increasing the quitting period [21].

Unfortunately, a significant positive correlation between students’ risk factors and their intentions to change could not be detected. These results could be inferred from the implication of COVID-19 restrictions’ experience and its health-related effects. As risk perceptions not only reflect the personal information but also personal experiences [19].

The effective implementation of lifestyle medicine, the therapeutic use of lifestyle interventions on health, can effectively reduce the overall risk for cardiovascular morbidity and mortality [28]. Unfortunately, during the COVID-19 pandemic, Riyadh residents’ nutritional behaviors had been altered. Most of them ate homecooked meals of low quality and high quantity [29]. Moreover, the increased home quarantine time during the COVID-19 pandemic prevented the Saudi population from practicing their daily sports activities and increased the health risks of the sedentary lifestyle [30]. Coinciding with reducing curfew and returning to normal life, Saudi female students showed moderate to high intention to activate lifestyle medicine in their lives by exercising at least 2½ hours a week and eating at least five portions of fruit and vegetables a day.

## Conclusions

SCD risk perception has many components that interact and result in ideal/non-ideal decisions, thus unworried individuals who had low perceived risk scores are more interested in seeking health preventive behaviors and vice-versa. Despite the presence of some limitations in our study - being a cross-sectional, self-reported survey using a convenience sample, it sheds light on young individuals' SCD risk perception. This is needed for planning comprehensive care for students with CVD risk factors for the effective communication of SCD risk and adequate preventive health measures to continue and improve health behaviors in reducing CVD outcomes and mortality. Future prospective research with large sample size is needed to determine young Saudi women’s deliberative, affective, and experiential SCD risk perceptions, awareness, and its cause-effect relation.
